# The association of serum C-peptide with the risk of cardiovascular events: a meta-analysis and systematic review

**DOI:** 10.1186/s13098-023-01142-6

**Published:** 2023-08-11

**Authors:** Mitra Kazemi Jahromi, Hamid Ahmadirad, Sanaz Jamshidi, Hossein Farhadnejad, Ebrahim Mokhtari, Tahere Shahrokhtabar, Saeed Tavakkoli, Farshad Teymoori, Parvin Mirmiran

**Affiliations:** 1 Endocrinology and Metabolism Research Center, Hormozgan University of Medical Sciences, Bandar Abbas, Hormozgan, Iran; 2 Nutrition and Endocrine Research Center, Research Institute for Endocrine Sciences, Shahid Beheshti University of Medical Sciences, Tehran, Iran; 3 Imam Ali Hospital, Shiraz University of Medical Sciences, Kazerun, Iran; 4 Department of Community Nutrition, School of Nutrition and Food Sciences, Shiraz University of Medical Sciences, Shiraz, Iran; 5 Department of Nutrition, School of Public Health, Iran University of Medical Sciences, Tehran, Iran

**Keywords:** C-peptide, Cardiovascular diseases, Meta-analysis

## Abstract

**Background:**

C-peptide is considered a peptide with active function in the body, which can affect people’s health. However, the results of previous studies on the possible association of C-peptide with the risk of cardiometabolic disorders have not been fully understood. This systematic review and meta-analysis aimed to investigate the association between serum C-peptide level and the risk of cardiovascular disease (CVD) events.

**Methods:**

The various important databases, including PubMed, Scopus, and Web of Science, were searched comprehensively to November 2022 to identify the relevant studies. The HR(95% CI) or OR(95% CI) for observational studies were extracted and converted into log HR or log OR and their standard deviation(SD) was computed. A random-effects model with an inverse variance weighting method was conducted, to calculate the pooled effect size.

**Results:**

Sixteen observational studies, including one case-control study, eight cohort studies, and seven cross-sectional studies were included in the current meta-analysis. The sample size ranged from 90 to 7030, with an age range from 12 to 85 years. During the follow-up time (ranging from 5 to 17 years), 4852 CVD events occurred. Based on cohort and case-control studies, the pooled results showed no significant association between serum C-peptide with CVD events risk (RR = 1.02;95%CI:0.91–1.15, I^2^ = 34.7%; P-heterogeneity = 0.140). For cross-sectional studies, the pooled results indicated a positive association between serum C-peptide and the odds of CVD outcomes (OR = 1.35;95%CI:1.04–1.76, I^2^ = 83.6%; P-heterogeneity < 0.001).

**Conclusions:**

The pooled results of the current study suggested that C-peptide level was not related to the risk of CVD events in cohort studies, however, the meta-analysis of cross-sectional studies showed a significant association between C-peptide and an increased risk of CVD events.

**Supplementary Information:**

The online version contains supplementary material available at 10.1186/s13098-023-01142-6.

## Background

Cardiovascular diseases (CVDs) is a general term covering the conditions affecting the heart or vessels [1]. CVDs are the most prevalent diseases and the major cause of mortality globally. Although the reports demonstrated that the age-adjusted CVD mortality rate is decreased by 60% worldwide during the past 30 years, the prevalence of the disease remains mainly unchanged due to the rapid rise in the aging population [2]. Furthermore, depending on a recent study in Iran, CVDs as the first leading cause of mortality and disability-adjusted life years (DALYs) resulted in 46% of all deaths and 20-23% of the burden of disease [3]. Pancreatic beta cells secrete insulin and C-peptide equimolarly. However, C-peptide, as a 31-amino acid polypeptide, stays longer in the blood than insulin. These characteristics have caused serum C-peptide to be considered as a surrogate biomarker for investigating the insulin secretion status and the pancreatic beta cell’s function. Besides, C-peptide is now recognized as a peptide with active function in the body that can affect individuals’ health [4].

Previous studies have shown inconsistent findings on the possible association of C-peptide level with the risk of chronic diseases such as cardiometabolic disorders. A recent meta-analysis reported that low serum C-peptide level were significantly associated with increased incidence of coronary heart disease (CHD) and cerebral infarction. Also, this study showed that serum C-peptide is inversely associated with blood lipid level and promotes lipid deposition [5]. Chung et al. also demonstrated that serum C-peptide level has an inverse correlation with cardiovascular autonomic neuropathy among T2DM patients [6]. Furthermore, in some studies, C-peptide has been proposed as a therapeutic agent for the prevention of vascular damage in type 1 diabetes (T1DM) [7, 8]. However, in other studies, results have shown that higher levels of serum C-peptide are associated with arterial stiffness, CVDs, and all-cause mortality in non-diabetic patients [9–12]. Also, the studies conducted on diabetic patients showed that the increase in serum C-peptide levels can increase the risk of early atherosclerosis [13, 14]. Furthermore, another study showed that 2-h serum C-peptide level may be a risk factor for carotid intima-media thickness changes [15].

On the other hand, a recent retrospective cohort study found that the relationship between serum C-peptide level and cardiovascular risk is different depending on the history of T2DM. Unlike the participants without previous T2DM, the serum C-peptide level had a “V” shaped relationship with cardiovascular biomarkers, the associations of C‑peptide with these biomarkers and events were not significant in the patients with previous T2DM [4].

Considering the inconsistent results in the past studies on the association of C-peptide level and risk of cardiovascular disorders, the present meta-analysis was intended to be an effective step in the correct and clear understanding of the relationship between the serum C-peptide level and the risk of cardiovascular diseases by combining all the data and presenting a general result. The findings of this study can also be used as a basis for future research regarding the therapeutic use of C-peptide in cardiometabolic disorders.

## Method

### Literature research

To detect the published papers until November 2022, a comprehensive and systematic search was conducted through PubMed, Scopus, and Web of Science databases without any language, time, and study design limitations. The systematic search was carried out using the following keywords and MeSH terms: “C-Peptide” or “proinsulin” AND “cardiovascular” or “heart disease” or “vascular” or “stroke” or “cardiac” or “myocardial” or “coronary artery”. To prevent missing any related publications, we manually searched the reference lists of all obtained studies. The present systematic and meta-analysis was performed according to the Preferred Items for Systematic Review and Meta-Analyses (PRISMA) guidelines[16].

### Inclusion and exclusion criteria

Two reviewers independently scanned the title and abstract of all publications. The studies that met the following criteria were considered eligible for inclusion: (1) the original article, (2) adolescent and adult populations (3) cohort, cross-sectional or case-control design, and (4) reported Hazard Ratio (HR), Odds Ratio (OR), and Relative Risk (RR) with 95% Confidence Intervals (CIs). Studies with the following features were excluded: (1) Review, meta-analysis, and randomized clinical trial studies (2) unpublished data, (3) studies without sufficient data, and (4) congress abstracts, patents, dissertations, letters, comments, and conference papers.

### Data extraction

The following information was collected from eligible studies: author’s name, publication year, location of the study, design, number of participants and CVD events, age and gender of subjects, tools used for serum C-peptide measurement, outcomes, reported HR or OR with 95% CI for CVD events, adjusted covariates, and the duration of follow-up. Two investigators independently extracted the information to increase the accuracy of data collection.

### Validity and quality assessment of studies

The quantitative content evaluation of each article was conducted according to the STROBE statement checklist[17]. Two reviewers examined all qualified studies for methodological quality assessment, independently using the Newcastle–Ottawa Scales (NOS) designed for cohort, case-control, and cross-sectional studies. Included studies based on NOS scores were categorized into three groups low, moderate, and high-quality[18].

### Statistical analysis

This meta-analysis includes fourteen eligible papers which consist of sixteen studies (eight cohorts, seven cross-sectionals, and one case-control). The HR (95% CI) for cohorts and the OR (95% CI) for cross-sectional and case-control studies were extracted and converted into log HR, then their standard deviation (SD) was computed. A random-effects model with an inverse variance weighting method was conducted, to calculate the pooled effect size. The I^2^ quantity[19] and Cochran’s Q test [20] were used to measure and evaluate between-study heterogeneity. In the present meta-analysis, the between-study heterogeneity test was statistically significant, thus we carried out subgroup analysis according to the region and diabetes status for cohort and cross-sectional studies, to detect the potential heterogeneity sources using a fixed-effects model. Due to the small number of included studies, the meta-regression analysis was not conducted to evaluate the heterogeneity sources across studies. We examined possible publication bias using the visual assessment of funnel plots and results of Egger’s regression test, and Begg’s adjusted rank correlation test. The trim-and-fill method was used to estimate the potential missing studies. Also, sensitivity analysis was performed, by removing each study one by one, to explore the impact of each study and recalculating the pooled effect size after its exclusion. All analyses were conducted using STATA 11.2 software. In all analyses, the statistically substantial value was considered as p < 0.05. All statistical tests were two-sided.

## Results

### Literature search and studies characteristics

**Fig. 1:**
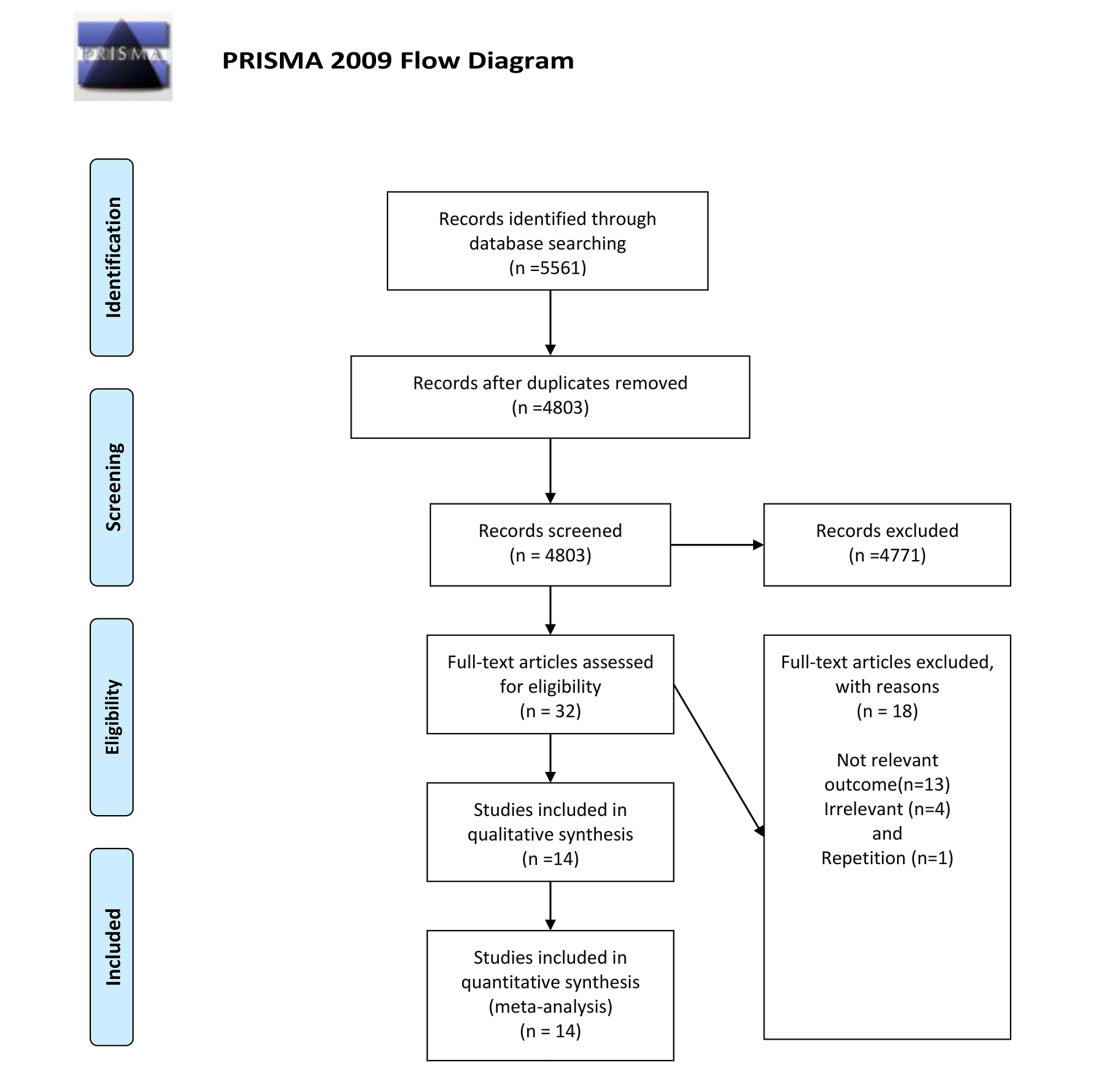
Flow diagram of selection of the published studies

The flow diagram of included studies is presented in Fig. 1. Initially, 5561 papers were obtained from the online database search. After scanning based on title and abstract, and removing duplicates (n = 758) and irrelevant articles (n = 4771), 32 papers potentially eligible were detected. After the full-text review, 18 papers were excluded due to not relevant outcomes (n = 13), not reported related data (n = 4), and repetition (n = 1). Finally, fourteen papers including 16 studies (cohort: n = 8, case-control: n = 1, and cross-sectional: n = 7) were included in the present meta-analysis.

The detailed characteristic of 16 studies is shown in Table 1. These studies were done between 1992 and 2021 and were performed in Italy (n = 5), the United States (n = 4), China (n = 3), Sweden (n = 2), Korea (n = 1), and the United Kingdom (n = 1). The sample size ranged from 90 to 7030, with an age range from 12 to 85 years. During the follow-up time (ranging from 5 to 17 years), 4852 CVD events occurred (Panero et al. study did not report the number of events). All studies were done on both genders except Dong et al. study which was done exclusively on female participants. All cohort studies reported the measure of association as HR and 95% CI, and one case–control and seven cross-sectional studies reported the OR and 95% CI. Most studies adjusted the association for some conventional risk factors, including age, sex, BMI, diabetes status, smoking, and alcohol drinking, however, three studies do not control any risk factors. Many included studies used laboratory analyses, radioimmunoassay, and the chemoluminescence method to measure serum C-peptide levels. All studies had high quality according to the NOS criteria for cohort, cross-sectional, and case-control studies. Quality assessments of studies are provided in Supplementary Table S1.

**Table 1:** Characteristics of included studies in the meta-analysis

Studies	Country	Study design	Sample size/Cases	Gender, age range	Exposure assessment	Outcome	comparison	HR or OR (95% CI)	Adjustment for covariate	Follow up (years)	NOS scores
Faglia,E.2002	Italy	Cohort	735/42	M,F 40–65 years	-	Cardiac events	C-peptide (nmol/l)	HR= 0.99 (0.96– 1.04)	-	5 years	8/9
Pikkemaat,M.2019	Sweden	Cohort	398/40	M,F 52.4 ± 8.7 years	Laboratory analyses	Ischemic stroke events	C-peptide (Per 1 nmol/ l)	HR= 1.22 (0.62– 2.39)	Age, sex, smoking, systolic blood pressure, HbA1c, antihypertensive treatment, BMI, CRP, eGFR, cholesterol, and previous myocardial infarction or ischemic stroke	17 years	8/9
Pikkemaat,M.2019	Sweden	Cohort	398/51	M,F 52.4 ± 8.7 years	Laboratory analyses	Myocardial infarction events	C-peptide (Per 1 nmol/ l)	HR= 1.47 (0.84– 2.58)	Age, sex, smoking, systolic blood pressure, HbA1c, antihypertensive treatment, BMI, CRP, eGFR, cholesterol, and previous myocardial infarction or ischemic stroke	17 years	8/9
Schrauben,S.2019	U.S	Cohort	1883/220	M, F 56.5 ± 11.9 years	NA	Atherosclerotic CVD events	C-peptide Per 1 SD	HR = 1.02 (0.84–1.24)	Age, sex, race, ethnicity, level of education, clinical center, BMI, waist circumference, smoking status, systolic BP, ACEi/ARB use, HDL, LDL, triglycerides, high sensitivity CRP, fat-free mass, eGFR, hemoglobin, physical activity, use of statins, use of other lipid-lowering medications, history of CVD, 24-h urine protein, FGF-23, uric acid, serum albumin	7.7years	9/9
Bo, S.2012	Italy	Cohort	2113/278	M, F 66.9 ± 10.2 years	Enzyme immunoassay	CVD events	C-peptide (T3 vs. T1)	HR = 0.93 (0.68–1.27)	Age, sex, BMI, smoking, time since diagnosis, insulin treatment, values of HbA1c, systolic blood pressure, HDL-cholesterol and triglycerides, presence of retinopathy, nephropathy, neuropathy and cardiovascular diseases	14years	9/9
Panero,F.2009	Italy	Cohort	471/NA	M, F 38.6 ± 9.4 years	NA	Macro vascular complications events	C-peptide (nmol/l)	HR = 0.77 (0.38–1.58)	Age, sex, diabetes duration, individual cumulative A1C average during the study period, hypertension, and cardiovascular diseases	10years	8/9
Panero,F.2009	Italy	Cohort	471/242	M, F 38.6 ± 9.4 years	NA	Micro vascular complications events	C-peptide (nmol/l)	HR = 0.59 (0.37–0.94)	Age, sex, diabetes duration, individual cumulative A1C average during the study period, hypertension, and cardiovascular diseases	10years	8/9
Koska,J.2021	U.S	Cohort	1693/479	M-F, 60 ± 9 years	Radioimmunoassay	CVD events	C-peptide (nmol/l)	HR = 1.27 (1.00–1.63)	Glucose lowering group, clinical and demographic risk factors, baseline insulin and sulphonylureas, severe hypoglycaemia.	5.3years	9/9
Dong, M.2013	China	Case–control	Control = 60 Case = 30	F 69.4 years	Chemoluminescence method	Acute myocardial infarction events	C-peptide (ng/ml)	OR = 1.45 (0.55–3.82)	Oestrone, oestradiol, testosterone, WHR, BMI, hypertension, diabetes and SHBG	-	8/9
Winocour,P.1992	UK	Cross-sectional	90/23	M, F 17–70 years	Radioimmunoassay	CHD events	C-peptide (nmol/l)	OR = 1.98 (0.13–29.3)	NA	-	5/7
Chen,Z.2021	U.S	Cross-sectional	3752/1832	M-F, 60.2 ± 13.1 years	Radioimmunoassay	SC-MI events	C-peptide (Q4 vs. Q1)	OR = 1.48 (1.18–1.87)	Age, gender, race, smoking, drinking, physical activity, BMI, TC, triglycerides, CRP, creatinine, glucose, glycated hemoglobin, and insulin	-	7/7
Li,W.2014	U.S	Cross-sectional	7030/143	M-F, 12–85 years	Radioimmunoassay	Stroke events	C-peptide (nmol/L)	OR = 3.71 (1.78–7.75)	Age, sex, ethnicity, height, weight, education level, physical activity, smoking status, alcohol use, HDL, LDL, triglycerides.	-	7/7
Wang,Y.2019	China	Cross-sectional	3143/ 936	M, F > 18 years	Immunoassay	CVD events	C-peptide (Q4 vs. Q1)	OR = 1.27 (1.13–1.42)	Age, gender, diabetes duration, current smoking, HbA1c, dyslipidemia, BMI, hypertension, use of insulin, statins, insulin-secreting agents.	-	7/7
Pontiroli,A.1997	Italy	Cross-sectional	93/54	M-F, 59–77 years	Radioimmunoassay	CHD events	C-peptide per 1 unit	OR = 1.47 (1.15–1.86)	-	-	5/7
Chung,J.2019	Korea	Cross-sectional	939/191	M-F, 62.6 years	Radioimmunoassay	CAN events	C-peptide (nmol/l)	OR = 0.67(0.52–0.87)	Age, gender, BMI, hypertension, hyperlipidemia, hemoglobin A1C, diabetes duration, CRP, anti-diabetic therapy UACR and eGFR.	-	7/7
Wang,L.2015	China	Cross-sectional	501/291	M-F 67.0 ± 8.0 years	Chemiluminesence immunoassay	CAD events	C-peptide (ng/mL)	OR = 1.51 (1.13–2.01)	Age, gender, BMI, duration of T2DM, history of insulin secretagogues treatment, insulin treatment and other anti-diabetic medications, fasting glucose, fasting plasma insulin, hypoglycaemic episodes, smoking and drinking statuses, UA, eGFR, hypertension and dyslipidemia UA, uric acid; eGFR, estimated glomerular filtration rate	-	6/7

Abbreviations:

BMI, body mass index; CAD, Coronary artery disease; CAN, Cardiovascular autonomic neuropathy; CI, confidence interval; CRP, C-reactive protein; CHD, coronary heart disease ;CVD, cardiovascular disease; HDL, high density lipoprotein; HR, hazard ratio; HbA1c, hemoglobin A1C; GFR, glomerular filtration rate; LDL, low density lipoprotein; OR, odds ratio; NOS, Newcastle-Ottawa scale; M, Male; F, Female; SC-MI, Subclinical myocardial injury; SD, Standard Deviation; T, tertile

### Meta-analysis on the association between C-peptide and CVD events

**Fig. 2:**
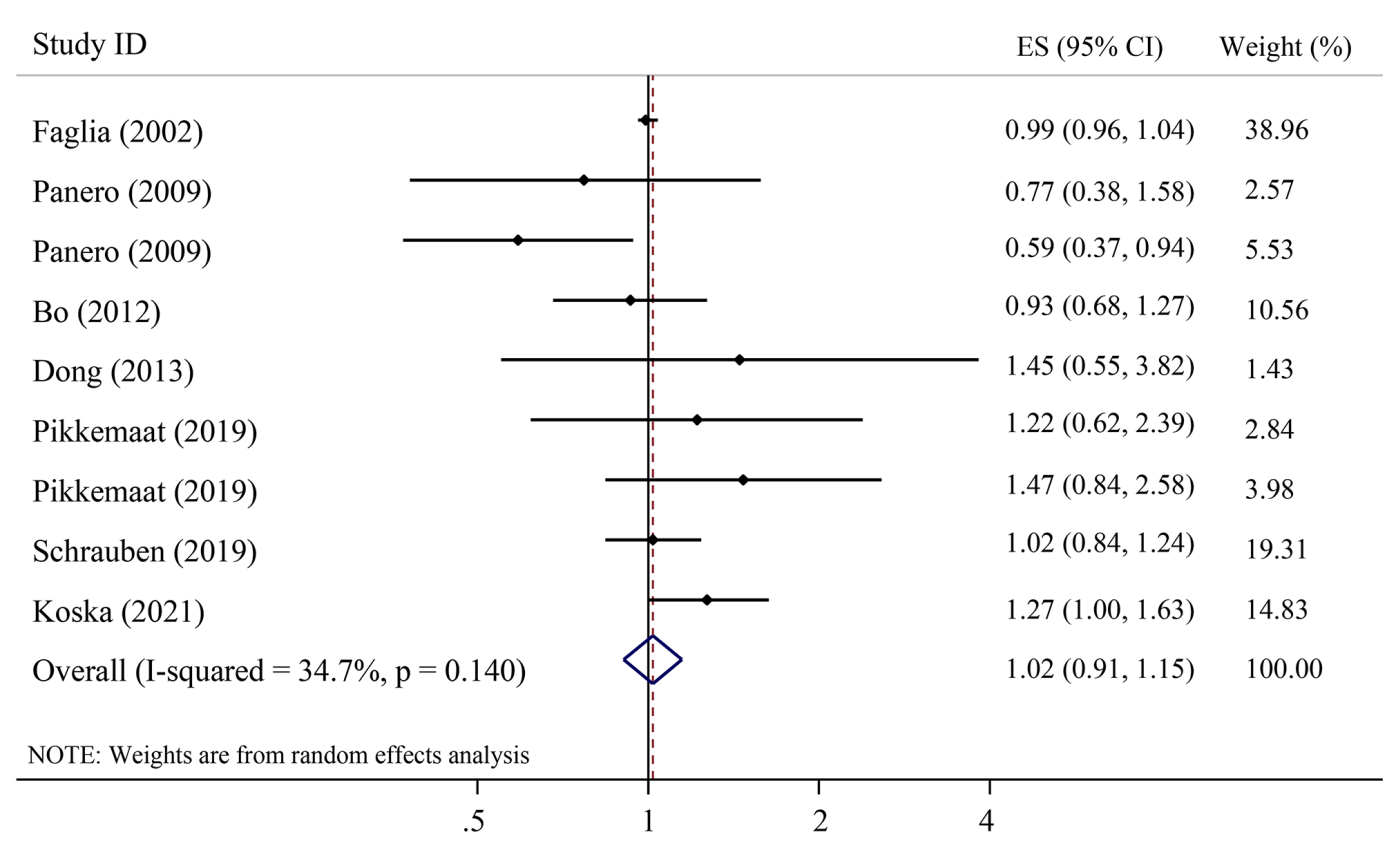
The association between serum C-peptide levels with risk of cardiovascular diseases event in cohort studies

Figure 2 indicated the overall summary estimate of HR (95% CI) for the association between serum C-peptide levels and the risk of CVD events in a case-control and eight cohort studies. The pooled RR calculated using the random-effects model indicated no significant association between serum C-peptide levels with the risk of CVD events (RR = 1.02; 95% CI: 0.91–1.15). Also, there was no significant heterogeneity between studies (I^2^ = 34.7%; P-heterogeneity = 0.140).

**Fig. 3:**
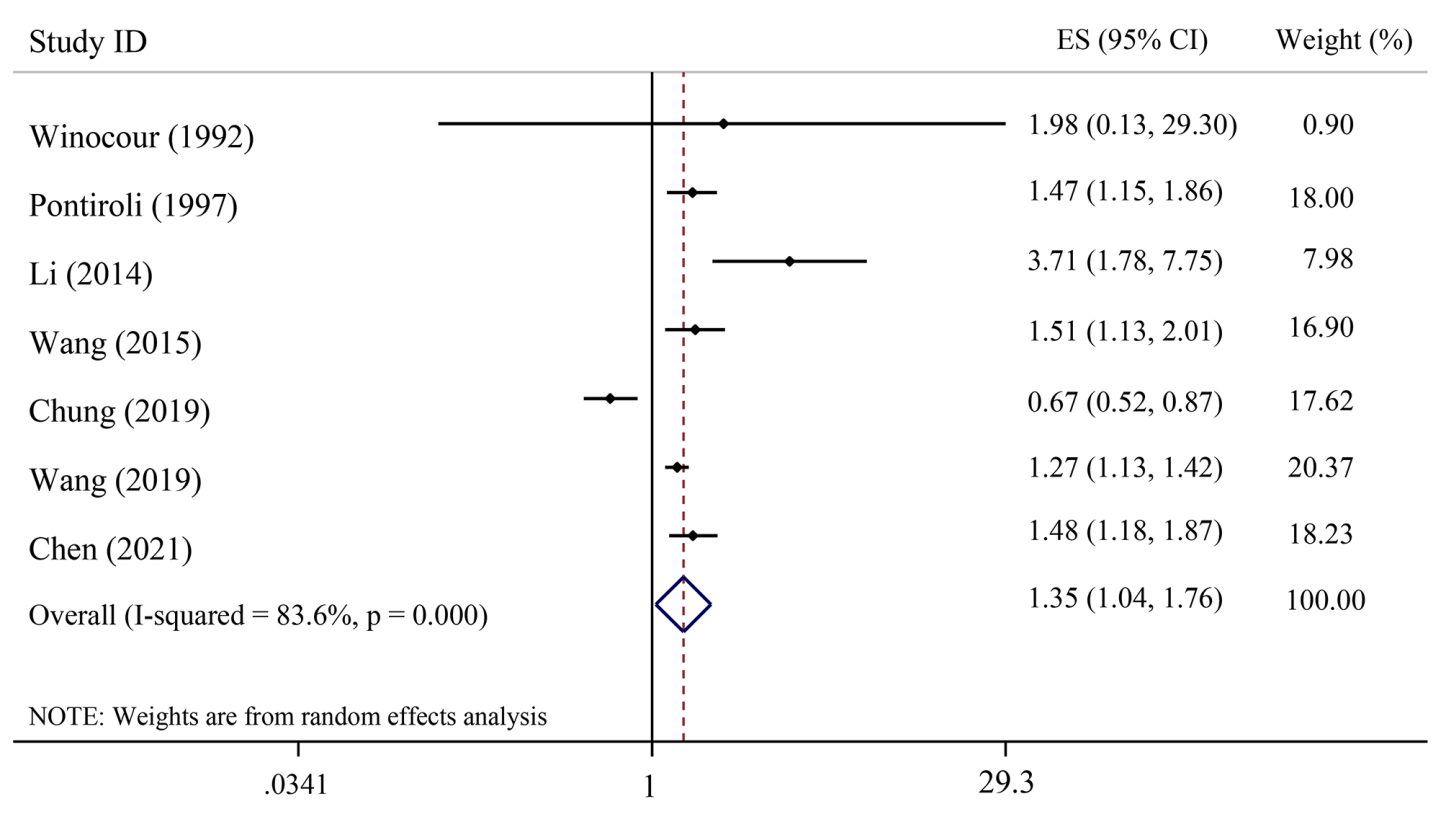
The association between serum C-peptide levels with risk of cardiovascular disease event in the cross-sectional studies

The association of serum C-peptide levels with the odds of CVDs in cross-sectional studies is presented in Fig. 3. The pooled OR using a random-effects model showed a significant positive relation between serum C-peptide and the odds of CVDs (OR = 1.35; 95% CI: 1.04–1.76), also there is considerable heterogeneity between studies (I^2^ = 83.6%; P-heterogeneity < 0.001).

Table 2 showed the findings of subgroup analysis for addressing the source of heterogeneity based on region and participants’ diabetes status in the cohort and cross-sectional analyses. Subgroup analysis in cohort studies demonstrated that serum C-peptide-CVDs event relationship was not significant in each subgroup of the region (Europe: n = 6, other: n = 3) and diabetes status (diabetic population: n = 7, general population: n = 2). Also, we observed any significant between-subgroups heterogeneity for the region and diabetes status.

**Table 2:** Summary relative risk (RR) estimates [95% confidence intervals (CIs)] for sub-group analysis of the association between the serum c-peptide levels with the risk of cardiovascular diseases events

Subgroups	Study numbers	Summary RR (95% CI)	Between studies			Between subgroups	
			I^2^	P_heterogeneity_		Q	P_heterogeneity_
Cohort and case-control studies Region						2.45	0.117
Europe	6	0.99 (0.95–1.03)	34.4	0.178			
Other	3	1.12 (0.96–1.30)	8.1	0.334			
Diabetes status						0.16	0.688
Diabetic population	7	0.99 (0.96–1.03)	48.3	0.078			
General population	2	1.03 (0.85–1.25)	0.0	0.480			
Cross-sectional studies							
Region						7.99	0.018
Asia	3	1.18 (1.07–1.30)	91.3	< 0.001			
America	2	1.61 (1.29–2.00)	81.7	0.019			
Europe	2	1.47 (1.16–1.87)	0.0	0.830			
Diabetes status						5.18	0.023
Diabetic population	5	1.22 (1.11–1.33)	84.5	< 0.001			
General population	2	1.61 (1.29–2.00)	81.7	0.019			

All statistical tests were two-sided

However, in cross-sectional studies that subgroup analyses conducted on the region (Europe: n = 2, America: n = 2 Asia: n = 3) and participants’ diabetes status(diabetic population: n = 5, general population: n = 2), a positive significant relationship in Asia (RR = 1.18; 95% CI: 1.07–1.30), America (RR = 1.61; 95% CI: 1.29–2.00) and Europe (RR = 1.47; 95% CI: 1.16–1.87) were observed, also there is significant heterogeneity between region subgroups (Q statistic = 7.99; P-heterogeneity = 0.018).

In diabetes status subgroups, there is a positive significant association among the diabetic population (RR = 1.22; 95% CI: 1.11–1.33) and the general population (RR = 1.61; 95% CI: 1.29–2.00), also a significant heterogeneity between diabetes status subgroups (Q statistic = 5.18; P-heterogeneity = 0.023) was detected. Regarding Table 2, region and diabetes status are considered the sources of heterogeneity between subgroups in cross-sectional studies.

### Publication bias

According to visual inspection of the funnel plot (Fig. 3sup, A), Begg’s test (P-value = 0.677), and Egger’s test (P-value = 0.689) there is no publication bias for studies examining the association between serum C-peptide with CVD events in cohort studies. Also, for cross-sectional studies, based on the P-values of Begg’s (0.881) and Egger’s test (0.619) there was no publication bias. Begg’s funnel plot for cohort and cross-sectional studies is presented in Fig S1.

### Sensitivity analysis

To explore each study’s impact on the pooled effect size, we used a random-effects model. After removing each study, we find out any significant change in the pooled effect size of the association between serum C-peptide levels with CVD in the meta-analysis on the cohort (0.98–1.03) and cross-sectional (1.23–1.46) studies. Findings showed that cohort studies were not affected by each included study, however, pooled OR of the cross-sectional meta-analysis becomes non-significant by omitting each included study except for studies conducted by Winocour et al. and Chung et al.

We have two studies that have not adjusted the effect of the confounder variable. To compute the pooled RR for adjusted studies, we conducted subgroup analysis and omitted Faglia et al. study and Winocour et al. study in the cohort and cross-sectional study, respectively. There is no significant change in RR obtained from subgroup analysis in the cohort (RR = 1.02; 95% CI: 0.85–1.23) and cross-sectional (RR = 1.34; 95% CI: 1.02–1.75) versus their overall pooled RR.

## Discussion

The findings from this meta-analysis systematically reviewed fourteen studies, which examined the association between C-peptide level and CVD events. Our results revealed no significant association between serum C-peptide levels with the risk of CVD events in a case-control and eight cohort studies. However, a significant positive relation between serum C-peptide and the odds of CVDs was seen in cross-sectional studies. Also, the results of the subgroup analysis based on the region and diabetes status indicated no noticeable association between serum C-peptide and CVDs in studies with case-control and cohort design. Nevertheless, cross-sectional studies conducted in Asia, America, and Europe and those studies in the diabetic and general populations had a significant association between C-peptide level and CVD events.

In line with our findings from pooled HR of cohort studies, a meta-analysis by Autier et al. revealed no evidence of an association between breast cancer risk and serum C-peptide values [21]. Most of the included studies in our meta-analysis found an insignificant association between C-peptide and CVD events. However, Panero et al. investigation reported the protective effect of plasma C-peptide levels on the incidence of microvascular complications risk in type 1 diabetic participants [22]. Although evidence in this area is still quite limited, C-peptide would activate endothelial NO synthase and Na^+^/K^+^-ATPase enzymes by interacting with membrane receptor on a molecular level which might have a favorable impact on both diabetic nephropathy and neuropathy. Also, C-peptide enhances skeletal muscle and skin blood flow, augments nerve function, diminishes urinary albumin excretion, and decreases glomerular hyperfiltration, only in individuals who suffer from type 1 diabetes and lack of C-peptide, and not in those with healthy status [23]. In contrast, Pikkemaat et al. showed that 1 nmol/l increase in C-peptide concentration is associated with all-cause death, underlying cardiovascular death. However, inadequate control for adiposity, different target groups (patients with type 2 diabetes), and limited sample size in this study should be mentioned [24]. It has been suggested that C-peptide function in type 1 and 2 diabetes mellitus are contrasting. For instance, due to the lack of insulin and C-peptide secretion in type 1 diabetes, it has been proposed that C-peptide can directly reduce lesions, apply anti-fibrotic and anti-apoptotic effects, and perform as a protective marker. However, in type 2 diabetes, C-peptide elevations cause insulin resistance consequence [25]. Since our findings include the pooled results from studies conducted on type 1 diabetes [22], type 2 diabetes [24–26], and chronic kidney disease [27], reliance on the results should be done with caution. Moreover, in Koska et al. study, a U-shaped relationship between C-peptide and CVD risk was observed, even so, the typical participants in this study was male with known/at high risk for CVD. Therefore, the findings might not be generalizable [26].

Our findings on the association between serum C-peptide and CVDs based on the cross-sectional studies are similar to some of the previous meta-analyses claiming that greater circulating C-peptide level could be a predictive marker for chronic diseases risk susceptibility, such as various cancer [28, 29]. Another meta-analysis reported increased risks of pancreatic and colorectal cancers associated with higher levels of C-peptide concentration [30]. It has been suggested that C-peptide can act as a suppressor of reactive oxygen species formation in endothelial cells and a stimulator of various neurotrophic factors including neurotrophin and nerve growth factors [31, 32]. Also, Thota et al. have remarked in a meta-analysis of observational studies that obese participants had a higher pooled mean net of total C-peptide concentration compared to those non-obese [33]. C-peptide role in promoting atherogenesis thus increasing the risk for CVD may contribute to inducing inflammatory markers infiltration and migration of vascular smooth muscle cells [34, 35]. On the other hand, C-peptide had a considerable correlation with some aspects of metabolic syndrome including high-density lipoprotein-cholesterol (HDL-C), waist circumference, and uric acid [36]. As Li et al. claimed, serum C-peptide had a strong negative association with HDL-C levels [37]. Based on these findings, it can be assumed that the C-peptide concentrations’ effect on CVD mortality can be originated from, at least in part, by metabolic disturbances.

It should be noted that since some evidence believed that dietary patterns with higher amounts of whole grains, vegetables, fruits, fiber, and coffee intake might be associated with lower plasma C-peptide level, while higher levels of refined grains and animal products might be associated with higher C-peptide level [38], evaluation of dietary patterns are valuable, which the lack of this assessment can be seen in the included studies. In contrast to our results, a previously conducted meta-analysis by Qin and colleagues showed that low serum C-peptide concentration increases lipid deposition, and accelerates the incidence of diabetes, cerebral infarction, and coronary heart disease [5]. Since the mentioned meta-analysis only includes seven studies, mostly domestic studies with small sample sizes, more research in the future are needed. Similarly, Chung et al., reported that circulating C-peptide level was inversely associated with cardiovascular autonomic neuropathy in individuals with type 2 diabetes [6]. In total, these findings are highly contradictory and require further investigation. We failed to find significant results in subgroup analyses of cohort studies, however, subgroup analyses conducted in cross-sectional studies showed a positive significant association between C-peptide and CVD events in Asia, America, and Europe, and among the diabetic population and the general population.

One of the reasons that can explain the non-significant relationship between C-peptide and CVD events based on the pooled results of cohort studies is that, in most of these studies, serum C-peptide has been measured as the main exposure of the study only at one point (at the beginning of the study), while it is possible that the baseline level of C-peptide of the individuals at the beginning of cohort studies did not reflect its level in the participants during the long term follow-up, because the C-peptide level may have changed in the study population during follow-up. So, if serum C-peptide was measured at several points in time in these cohort studies, it more could help determine the serum C-peptide accurately in participants and determine its real relationship with the risk of CVD events in them. Also, considering that cross-sectional studies cannot show the real causality, the pooled results reported in these studies on the significant relationship between high serum C-peptide level and increased risk of CVD events may be somewhat unrealistic; considering that, in cross-sectional studies, the levels of exposure (serum C-peptide) and outcome (CVD events) for individuals have been determined at a specific time point, it is possible that, the serum C-peptide level of patients with CVD events has subsequently increased due to their inappropriate and unstable metabolic condition, such as insulin resistance, metabolic syndrome, obesity, etc., and therefore, the high serum C-peptide level maybe not a definite risk factor for CVD events in individuals.

The current study has several noteworthy strengths. To our knowledge, our study is the first meta-analysis investigating this topic with large sample size. However, a few limitations in this study should be kept in mind. Although the pooled results of cross-sectional studies were found to be remarkably significant, it had weak sensitivity and by discarding any of the 5 articles that reported significant findings from the analysis, the OR value became non-significant. In addition, based on the publication bias assessment, if the two missing studies were included in the analyses, the cross-sectional results would be insignificant. Also, different methods were used to assess circulating C-peptide levels. Moreover, included studies evaluated various micro and macrovascular complications which results in high heterogeneity. It seems that more studies are needed to evaluate our hypothesis.

## Conclusion

The evidence presented in our study indicates no significant association between C-peptide and an increased risk of CVD events in cohort studies, while a meta-analysis of studies with the cross-sectional design suggested that the higher C-Peptide level is associated with the increased risk of CVD events. Additional investigations are needed to shed light on this issue.

### Electronic supplementary material

Below is the link to the electronic supplementary material.

Supplementary Material 1

## Data Availability

The data used and/ or analyzed in the present study is available from the corresponding author on reasonable request.
