# Supplementary material for: The association of serum C-peptide with the risk of cardiovascular events: a meta-analysis and systematic review
Source: Diabetol Metab Syndr. 2023 Aug 11;15:168. doi: 10.1186/s13098-023-01142-6 (PMC10416519; doi:10.1186/s13098-023-01142-6)
Supplement: Supplementary file 1 — Supplementary Material 1 [file 13098_2023_1142_MOESM1_ESM.docx]

**LIST OF SUPPLEMENTARY MATERIALS**

- **Table S1: The PICO criteria used for the present systematic review and meta-analysis**
- **Table S2: Search Strategies used for different databases**
- **Table S3:** **Quality assessment using New Castle - Ottawa Scale for cohort studies.**
- **Table S4: Quality assessment using New Castle - Ottawa Scale for case-control studies.**
- **Table S5: Quality assessment using New Castle - Ottawa Scale for cross sectional studies.**
- **Figures S1:** **Begg’s funnel plot (with pseudo 95% confidence interval) depicting log HR (hazard ratio) and OR (odds ratio) against their corresponding standard error for assessing the presence of publication bias in studies that investigated the association between serum C-peptide with cardiovascular diseases in the cohort (Fig A) and cross-sectional (Fig B) studies, respectively.**
- **Preferred Reporting Items for Systematic Reviews and Meta-Analyses (PRISMA)**

**Table S1. The PICO criteria used for the present systematic review and meta-analysis**

| **PICO criteria** | **Description** |
| --- | --- |
| **Patients** | Adult subjects |
| **Exposure** | Serum C-peptide level |
| **Comparison** | Highest category of C-peptide level versus the lowest category  and per 1 SD or unit of C-peptide |
| **Outcome** | Cardiovascular diseases events |

**Table S2: Search Strategies used for different databases**

| **Database** | **Search strategy** | **Number of publications** |
| --- | --- | --- |
| **PubMed** | (((cardiovascular*[tiab] OR vascular*[ti] OR stroke*[ti] OR cardiac*[ti] OR heart disease*[tiab] OR coronary artery*[tiab]) OR ("Heart Diseases"[Mesh]))) AND ("C-Peptide"[Mesh] OR proinsulin*[tiab]) | 691 |
| **Scopus** | ( ( TITLE-ABS-KEY ( "heart diseases" ) ) OR ( TITLE-ABS-KEY ( cardiovascular* ) ) OR ( TITLE-ABS-KEY ( stroke* ) ) OR ( TITLE-ABS-KEY ( "coronary artery" ) ) OR ( TITLE-ABS-KEY ( cardiac* ) ) OR ( TITLE-ABS-KEY ( myocardial* ) ) OR ( TITLE-ABS-KEY ( vascular* ) ) OR ( TITLE-ABS-KEY ( CVD ) ) ) AND ( ( TITLE-ABS-KEY ( ( c-peptide* ) ) OR TITLE-ABS-KEY ( ( proinsulin* ) ) ) ) | 2915 |
| **Web of**  **Science** | (TS=(stroke* OR CVD* OR cardiovascular* OR coronary artery* OR heart disease* OR myocardial* OR cardiac* OR vascular*)) AND TS=( c-peptide* OR proinsulin*) | 1955 |

**Table S3:** Quality assessment using New Castle - Ottawa Scale for cohort studies^*^

|  | **Selection** | | |  | **Comparability** | **Outcome** | | | Study score |
| --- | --- | --- | --- | --- | --- | --- | --- | --- | --- |
| **Study** | Representativeness of  The exposed cohort | Selection of the non-Exposed cohort | Ascertainment of exposure | Demonstration that  The outcome of  Interest was not  Present at start of The study | Comparability of  Cohorts on the basis of design or analysis | Assessment of outcome | Was follow‐ up  Long enough  For the outcome to occur? | Adequacy of follow up of  cohorts |  |
| Pikkemaat et al, 2019 | * | * | * | * | ** | * | * | * | 8/9 |
| Bo et al, 2012 | * | * | * | * | ** | * | * | * | 9/9 |
| Panero et al, 2009 | * | * | * | * | ** | * | - | * | 8/9 |
| Faglia et al, 2002 | * | * | - | * | ** | * | * | * | 8/9 |
| Koska et al, 2021 | * | * | * | * | ** | * | * | * | 9/9 |
| Schrauben et al, 2019 | * | * | * | * | ** | * | * | * | 9/9 |

** Study score less than 4 indicates low quality, a score of 4 to 6 represents moderate, and a score of more than 6 indicates as a high-quality*

**Table S4:** Quality assessment using New Castle - Ottawa Scale for case-control studies^*^

|  | **Selection** | | |  | **Comparability** | **Exposure** | | | **Study score** | **Quality of study** |
| --- | --- | --- | --- | --- | --- | --- | --- | --- | --- | --- |
| **First author (Year)** | Is the case definition adequate? | Representativeness of the cases | Selection of Controls | Definition of Controls | Comparability of cases and controls on the basis of the design or analysis | Ascertainment of exposure | Same method of ascertainment for cases and controls | Non-Response rate |  |  |
| Dong,2013 | * | * | * | * | ** | * | * | - | 8/9 | high |

*^*^ Study score less than 4 indicates low quality,a score of 4 to 6 represents moderate, and a score of more than 6 indicates as a high-quality*

**Table S5:** Quality assessment using New Castle - Ottawa Scale for cross sectional studies.

|  | **Selection** | | | **Comparability** | **Outcome** | | Study  score |
| --- | --- | --- | --- | --- | --- | --- | --- |
| Study | Representativeness of The exposed sample | Selection of the non-Exposed sample | Ascertainment of exposure | Comparability of Outcome groups on the  Basis of design or analysis | Assessment of outcome | Statistical test is appropriate |  |
| Winocour,1992 | * | * | - | ** | - | * | 5/7 |
| Pontiroli,1997 | * | * | - | ** | - | * | 5/7 |
| Li, 2014 | * | * | * | ** | * | * | 7/7 |
| Wang,2015 | * | * | * | ** | - | * | 6/7 |
| Wang,2019 | * | * | * | ** | * | * | 7/7 |
| Chung,2019 | * | * | * | ** | * | * | 7/7 |
| Chen,2021 | * | * | * | ** | * | * | 7/7 |

*†We used a modified NOS scale for cross sectional studies. Note: A score of 7 or higher was considered as good study (see Supplementary TextS3 for references and original and modified scales)*

**Figures S1:** **Begg’s funnel plot (with pseudo 95% confidence interval) depicting log HR (hazard ratio) and OR (odds ratio) against their corresponding standard error for assessing the presence of publication bias in studies that investigated the association between serum C-peptide with cardiovascular diseases in the cohort (Fig A) and cross-sectional (Fig B) studies, respectively.**


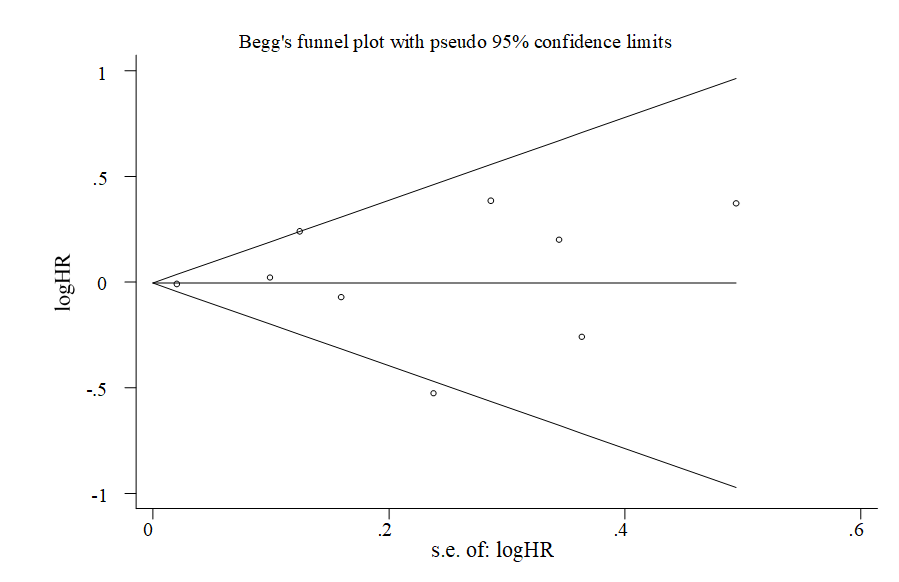
**Fig A.** **Fig B.**


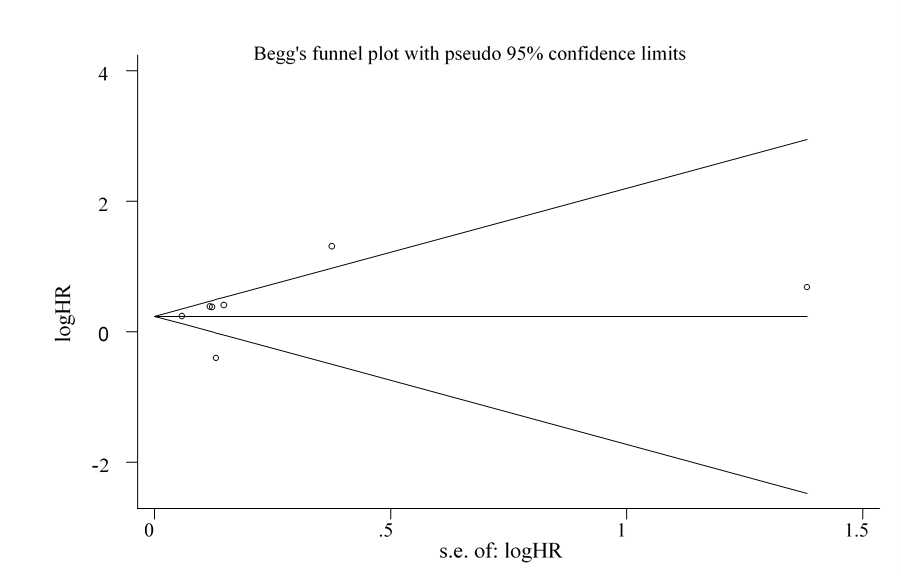


**PRISMA checklist**

| **Section/topic** | **#** | **Checklist item** | **Reported on page #** |
| --- | --- | --- | --- |
| **TITLE** | | |  |
| Title | 1 | Identify the report as a systematic review, meta-analysis, or both. | Page 1 |
| **ABSTRACT** | | |  |
| Structured summary | 2 | Provide a structured summary including, as applicable: background; objectives; data sources; study eligibility criteria, participants, and interventions; study appraisal and synthesis methods; results; limitations; conclusions and implications of key findings; systematic review registration number. | Page 2 |
| **INTRODUCTION** | | |  |
| Rationale | 3 | Describe the rationale for the review in the context of what is already known. | Pages 4, 5 |
| Objectives | 4 | Provide an explicit statement of questions being addressed with reference to participants, interventions, comparisons, outcomes, and study design (PICOS). | Pages 4, 5 |
| **METHODS** | | |  |
| Protocol and registration | 5 | Indicate if a review protocol exists, if and where it can be accessed (e.g., Web address), and, if available, provide registration information including registration number. | Page 5 |
| Eligibility criteria | 6 | Specify study characteristics (e.g., PICOS, length of follow-up) and report characteristics (e.g., years considered, language, publication status) used as criteria for eligibility, giving rationale. | Page 5 |
| Information sources | 7 | Describe all information sources (e.g., databases with dates of coverage, contact with study authors to identify additional studies) in the search and date last searched. | Page 5 |
| Search | 8 | Present full electronic search strategy for at least one database, including any limits used, such that it could be repeated. | Page 5 |
| Study selection | 9 | State the process for selecting studies (i.e., screening, eligibility, included in systematic review, and, if applicable, included in the meta-analysis). | Page 6 |
| Data collection process | 10 | Describe method of data extraction from reports (e.g., piloted forms, independently, in duplicate) and any processes for obtaining and confirming data from investigators. | Pages 5,6 |
| Data items | 11 | List and define all variables for which data were sought (e.g., PICOS, funding sources) and any assumptions and simplifications made. | Pages 6,7 |
| Risk of bias in individual studies | 12 | Describe methods used for assessing risk of bias of individual studies (including specification of whether this was done at the study or outcome level), and how this information is to be used in any data synthesis. | Pages 6,7 |
| Summary measures | 13 | State the principal summary measures (e.g., risk ratio, difference in means). |  |
| Synthesis of results | 14 | Describe the methods of handling data and combining results of studies, if done, including measures of consistency (e.g., I^2^) for each meta-analysis. | Page 7 |

Page 1 of 2

| **Section/topic** | **#** | **Checklist item** | **Reported on page #** |
| --- | --- | --- | --- |
| Risk of bias across studies | 15 | Specify any assessment of risk of bias that may affect the cumulative evidence (e.g., publication bias, selective reporting within studies). | Pages 6,7 |
| Additional analyses | 16 | Describe methods of additional analyses (e.g., sensitivity or subgroup analyses, meta-regression), if done, indicating which were pre-specified. | Pages 6,7 |
| **RESULTS** | | |  |
| Study selection | 17 | Give numbers of studies screened, assessed for eligibility, and included in the review, with reasons for exclusions at each stage, ideally with a flow diagram. | Figure1 and page 7 |
| Study characteristics | 18 | For each study, present characteristics for which data were extracted (e.g., study size, PICOS, follow-up period) and provide the citations. | Table1 |
| Risk of bias within studies | 19 | Present data on risk of bias of each study and, if available, any outcome level assessment (see item 12). | Table2 |
| Results of individual studies | 20 | For all outcomes considered (benefits or harms), present, for each study: (a) simple summary data for each intervention group (b) effect estimates and confidence intervals, ideally with a forest plot. | Figure 2,3 |
| Synthesis of results | 21 | Present results of each meta-analysis done, including confidence intervals and measures of consistency. | Figure 2,3 |
| Risk of bias across studies | 22 | Present results of any assessment of risk of bias across studies (see Item 15). | Pages 9-10 |
| Additional analysis | 23 | Give results of additional analyses, if done (e.g., sensitivity or subgroup analyses, meta-regression [see Item 16]). | Pages 8-10 |
| **DISCUSSION** | | |  |
| Summary of evidence | 24 | Summarize the main findings including the strength of evidence for each main outcome; consider their relevance to key groups (e.g., healthcare providers, users, and policy makers). | Page 10 |
| Limitations | 25 | Discuss limitations at study and outcome level (e.g., risk of bias), and at review-level (e.g., incomplete retrieval of identified research, reporting bias). | Pages 11-13 |
| Conclusions | 26 | Provide a general interpretation of the results in the context of other evidence, and implications for future research. | Pages 11-13 |
| **FUNDING** | | |  |
| Funding | 27 | Describe sources of funding for the systematic review and other support (e.g., supply of data); role of funders for the systematic review. | Pages 14 |

*From:*  Moher D, Liberati A, Tetzlaff J, Altman DG, The PRISMA Group (2009). Preferred Reporting Items for Systematic Reviews and Meta-Analyses: The PRISMA Statement. PLoS Med 6(7): e1000097. doi:10.1371/journal.pmed1000097

For more information, visit: **www.prisma-statement.org**.

Page 2 of 2
